# Effectiveness of continuous versus pulsed short-wave diathermy in the management of knee osteoarthritis: A randomized pilot study

**DOI:** 10.22088/cjim.10.4.431

**Published:** 2019

**Authors:** Selin Ozen, Ekin B Doganci, Ayla Ozyuvali, Ayse Peyman Yalcin

**Affiliations:** 1 Başkent University Faculty of Medicine, Department of Physical and Rehabilitation Medicine, Ankara, Turkey; 2 Sungurlu State Hospital, Sungurlu, Turkey; 3 Ordu Devlet Hastanesi, Ordu, Turkey; 4 University Faculty of Medicine, Department of Physical and Rehabilitation Medicine, Ankara, Turkey

**Keywords:** Osteoarthritis, Gonarthrosis, Pain, Diathermy, Women, Visual analogue scale

## Abstract

**Background:**

Short-wave diathermy (SWD) is an electrotherapeutic modality used in the conservative treatment of knee osteoarthritis (KOA). Electromagnetic radiation delivered in continuous (cSWD) or pulse (pSWD) mode provides a deep heating effect on tissues. There is no consensus on outcomes of treatment with cSWD versus pSWD in KOA. The aim of this study was to compare the effects of cSWD versus pSWD on pain, functionality and walking distance in KOA.

**Methods:**

34 female patients aged 49-65 with KOA were randomized into two groups. A total of 27 patients completed the study. One group (n=11) was treated with cSWD, the other (n=16) with pSWD for three weeks. Patients were assessed before, after and at one month post therapy. Outcome measures included visual analogue scale (VAS) for knee pain, Western Ontario and Mcmaster University Osteoarthritis Index (WOMAC) and a six-minute walking test (6MWT).

**Results:**

Based on the minimal clinically important improvement (MCII), there was a reduction in VAS and WOMAC scores in both cSWD and pSWD groups post treatment (-37.3mm, 31.2mm respectively for VAS and 26%, 23% respectively for WOMAC) and at one month post treatment. There was no difference in pre and post treatment VAS for pain, WOMAC or 6MWT scores between the two groups. There was a small post treatment effect size on between- group 6MWT scores (Cohen’s d: 0.238).

**Conclusion:**

Both treatment options appear to be efficacious in reducing pain and improving functionality in KOA. There was no between-group difference. A larger study must be conducted to consolidate these findings.

Knee osteoarthritis (KOA) is one of the most common forms of arthritis in the Western world, with a prevalence of 10 to 15% in adults over 60 years of age (1, 2). Even though total knee arthroplasty (TKA) is the definitive treatment for advanced KOA, KOA can lead to chronic joint pain, muscle weakness and loss of function in the earlier stages of disease; often patients require conservative and medical treatment long before surgical intervention would be considered (3, 4). A recent study by Losina et al. underlined the fact that expanding TKA eligibility increases KOA related costs substantially, further reiterating the need for effective non operative treatment options (5). Short-wave di**athermy (SWD**) is one of the oldest forms of electrotherapeutic modalities traditionally used in the treatment of symptomatic KOA (6). 

In 1891, Nikola Tesla first noted that heat resulted from irradiation of tissue with high-frequency alternating current and pointed out its possible medical uses. In the 1930s SWD, its physical properties and its beneficial therapeutic uses became a popular topic of discussion (7). SWD provides electromagnetic radiation (typically at a frequency of 27.12 MHz), either in continuous (CSWD, thermic) or pulsed (PSWD, athermic) mode. It is generally believed that the increase in tissue temperature achieved using CSWD induces vasodilatation, an increase in cellular activity, pain threshold and soft tissue extensibility and a reduction in muscle spasm (6, 8). 

PSWD provides radiation in the form of pulse trains (9). PSWD is mostly preferred for its athermal effects. It is believed that PSWD also enhances cellular activity (10, 11), with its physiological effects including an increase in blood flow and a decrease in joint pain and stiffness, inflammation and edema (12). The perceived anti-inflammatory effects of PSWD on the synovium, and the possible link between thermic SWD, increased synovitis and worsening of cartilaginous degeneration, (8,9) has resulted in increased use of PSWD in the treatment of KOA over the past ten to fifteen years (13). 

Despite SWD being a well established part of the conservative treatment of knee osteoarthritis (KOA), the Osteoarthritis Research Society International guideline for the non-surgical management of KOA did not feature SWD (14). The reason for this maybe is that even though SWD treatment appears to be effective in decreasing pain (15) and increasing muscle strength (16) in patients with KOA, there is no the consensus on the outcomes of treatment using CSWD versus PSWD. 

A systematic review and meta-analysis of the studies comparing the effectiveness of CSWD to PSWD published by Laufer et al. in 2012 concluded that findings to date suggested that PSWD was of no benefit (17). However, they did acknowledge that larger studies with comparable samples, protocols and outcome measures were required in order to draw firmer conclusions. A more recent systematic review also concluded that SWD provided pain relief in KOA patients but that it did not improve physical function. However, in contrast to the previous review, robust subgroup analysis this time revealed that PSWD was superior to CSWD in reducing pain, especially in females (18). The aim of our study was to compare the effects of continuous (thermic) versus pulsed (athermic) SWD on pain, function and activity in women with KOA especially focusing on the treatment effects of SWD alone. 

## Methods

The study took place between January 2013 and January 2016. Thirty four age and body mass index (BMI) matched female patients presenting to the outpatient clinic between the ages of 49-65 with complaints of bilateral knee pain and a diagnosis of KOA according to the American College of Rheumatology clinical criteria for the classification of osteoarthritis of the knee (19) were randomized into one of two treatment groups (thermic SWD and athermic SWD) using simple random sampling. Three of the patients initially assigned to the thermic SWD group could not tolerate the heat sensation and so were reassigned to the athermic SWD group before treatment commencement. Only patients with radiographic evidence of grade 2-3 osteoarthritis according to the Kellgren – Lawrance (K-L) scale (20), diagnosed by the same Physical and Rehabilitation Medicine (PRM) specialist, were included in the study. Exclusion criteria: 1) physical therapy to the knee joint over the past six months 2) reduction of range of motion of the knee 3) presence of low back/hip/knee/ankle joint pathologies or symptoms of pain 4) presence of inflammatory arthropathy 5) history of knee trauma or knee intervention over the past six months 6) presence of metal implants, a cardiac pacemaker or malignancy. Those taking non steroidal anti-inflammatory drugs (NSAIDs) were switched to diclofenac 75mg slow release once daily one week prior to treatment for the duration of the study. Written informed consent was obtained from all study participants prior to commencement of the study.

**Physical Therapy Modality:** SWD electromagnetic radiation at a frequency of 27.12MHz was applied in continuous mode (CSWD, thermic) in group one and pulsed mode (PSWD, athermic) in group two. Treatment was administered by the same physiotherapists using the Curapuls 419 SWD machines (Enraf-Nonius, Delft, the Netherlands). No other physical therapy was given. Sessions lasted fifteen minutes, on five consecutive days per week for a total of three weeks. 

**Assessment of Treatment effects:** Patients were assessed before, after and in one month post treatment. All assessments were carried out by the same PRM physician blind to the treatment received and knee radiographs. The primary outcome measure was pain, measured using a visual analogue scale (VAS). The VAS provides a subjective, visual pain score from 0-100mm scored by the patient where 0mm is no pain and 100mm the worst pain imaginable. Secondary outcome measures included the Western Ontario and Mcmaster University Osteoarthritis Index (WOMAC) and a-six minute walking test (6MWT) as a functional test of walking ability and exercise capacity (21, 22). All 6MWTs were performed in the same ten meter long gymnasium. The WOMAC aims to evaluate clinically important, patient‐relevant changes in health status as a result of treatment intervention to the knee (21). 

Ethical approval for the project was obtained from the Ankara University Faculty of Medicine Ethics Committee (decision no 10-417-13) in accordance with ethical standards on human experimentation and with the Helsinki Declaration of 1975, as revised in 1983. No financial support was received for the project.

**Statistical Analysis:** Articles on adequate sample sizes for pilot studies was used and a sample size of between ten and thirty patients was aimed for (23). The data was analyzed using SPSS for Windows (IBM^®^ SPSS^® ^statistics version 22). Chi square and Fisher’s exact tests were used for the cross tabs of categorical date. 

The normal distribution and homogeneity of the continuous variables were evaluated using the Kolmogorov- Smirnov test and Levene’s test respectively. p<0.05 signified an abnormal distribution/ non-homogeneity in which case non - parametric tests were used for further analysis. The student’s T test was used when comparing between group of parametric data and Mann Whitney U test for non-parametric data. 

The Friedman test was used to evaluate within and between group VAS and WOMAC scores. ANOVA with repeated measurements was used to compare within group 6MWT and for all the patients regardless of treatment group. When a statistically significant result was obtained, the post hoc Bonferroni multiple comparison test was used to identify pair wise differences. 

Minimal clinically important differences (MCID) /improvement (MCII) for VAS, WOMAC and 6MWT were used when interpreting the data. The MCII for the VAS score for pain in KOA was -19.9mm (24). In rehabilitation intervention, effects larger than 12% of the baseline score can be used as the MCID in the WOMAC (25). Based on the work of Redelmeier et al. 54 meters is often used as the MCID for the 6MWT (26). Effect size was calculated using Cohen’s d where the values of d for small, medium, and large effects are 0.2, 0.5, and 0.8 respectively (27). 

## Results

Eleven (40.7%) patients were treated with CSWD (group 1), sixteen (59.3%) were treated with PSWD (group 2). A total of seven patients randomized to the treatment groups were excluded from the study. Three of the seven patients failed to complete the treatment protocol, four of the seven patients were unable to attend follow up assessment one month after treatment (figure 1). 

Baseline characteristics of both groups have been given in table one. There was no statistical difference between the groups in terms of age distribution and BMI (P>0.05). There was also no statistically significant difference in the distribution of patients in the CSWD versus the PSWD group who were using NSAIDs (P=0.411). In addition, there was no significant difference in the Kellgren Lawrence grading of either the right or left knee between those taking NSAIDs and those on no analgesics (P=0.710 and P=0.687 respectively). 

On assessment, there was no statistically significant difference in the distribution of patients with Kellgren Lawrence grade 2 and Kellgren Lawrence grade 3 of the right knee between the treatment groups (P=0.061), this was also the case for the left knee (P=0.224). Table 1 here.

Based on the MCII, there was an important reduction in VAS scores for pain following treatment with both CWSD and PSWD immediately after the course of treatment (-37.3 mm and -31.2 mm respectively) and in one-month follow up (table 2). 

In addition, there was a clinically important reduction in total WOMAC scores after treatment in both groups; the change in the mean WOMAC total score was 26% in the CSWD group and 23% in the PSWD group. The clinically important change in WOMAC persisted one-month follow up post treatment (table 3). 

No clinically important change in 6MWT was detected in either treatment group (table 4). There was no difference in pre and posttreatment VAS for pain, WOMAC or 6MWT scores between the two groups. When comparing the post treatment scores of the two groups to one another, there was a small effect size in the 6MWT with a Cohen’s d of 0.238. None of the patients reported any side effects of treatment. 

**Fig . 1:**
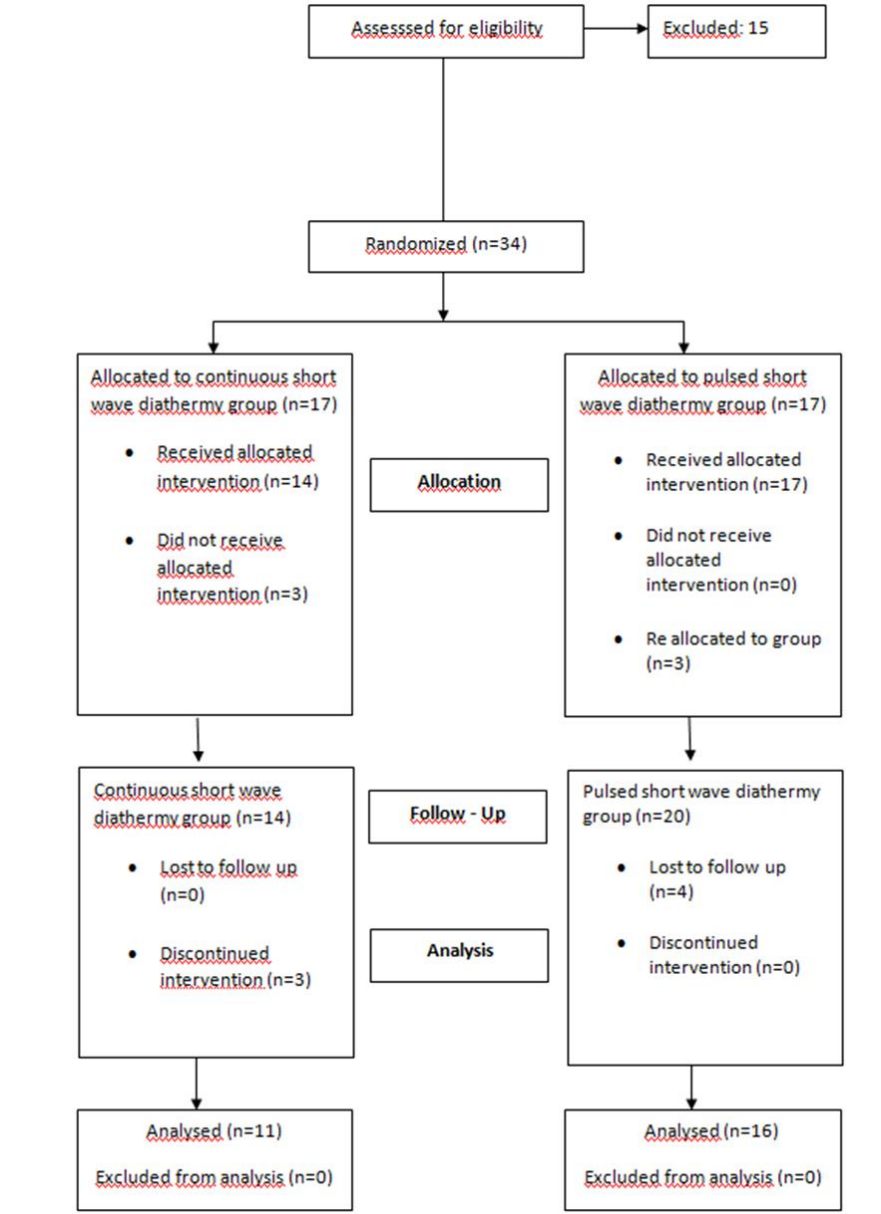
Patient enrollment flow chart

**Table 1:** Baseline patient characteristics

**Characteristics**	**Continuous SWD†** **N=11**	**Pulsed SWD†** **N=16** **N (%)**	**Total** **N=27** **N (%)**	**P (between groups)**
Age (years)Mean±SD Median; min- max	57.9±5.0 56; 52-65	54.8±4.4 56; 49-60	56.85±5.855 57; 49-65	0.900
BMI^α^ (kg/m^2^) Mean±SD Median; min- max	33.6±4.1 33.7; 26-40.6	34.7 ± 4.8 33.4; 28.9-42	34.2±4.5 33.7; 26-42	0.534
Symptom duration (months) Mean±SD Median; min- max	87.6±57.5 120.0; 4-180	36.6 ± 33.3 30;1-120	57.4 ± 50.6 48; 1-180	0.039
NSAIDs use, N (%)	5(45.5)	4(25)	9(33.3)	0.411
Right knee KL grade 2, N (%) Right knee KL grade 3, N (%)	4(36.4) 7(63.6)	12(75) 4(25)	16(59.3) 11(40.7)	0.061
Left knee KL grade 2, N (%) Left knee KL grade 3, N (%)	5(45.5) 6(54.5)	12(75) 4(25)	17(63) 10(37)	0.224
Pre treatment VAS* for pain (mm) Mean±SD Median; min- max	77.3±20.0 80; 50-100	75.0±21.6 80; 40-100	75.9±20.6 80; 40-100	0.839
Pre treatment 6MWT^◦^ (m) Mean±SD Median; min- max	352.3±67.4 350; 250-440	323.3±88.3 327.5; 80-460	335.07±80.343 340; 80-460	0.367
Pre treatment WOMAC¨ total score Mean±SD Median; min- max	52.1±18.1 54; 26-78	47.9±15.3 45; 16.73	49.6±16.3 48; 16-78	0.521

†Short-wave diathermy, ^α^Body mass index, ^∞^Kellgren Lawrance *Visual analogue scale for pain (range 0-100mm), ^◦^6 minute walking test, ¨ Western Ontario and Mcmaster University Osteoarthritis Index

**Table 2:** VAS scores before and after treatment in both groups

	**Continuous ** **SWD** **(n= 11)**	**Pulsed SWD** **(n= 16)**	**Total** **(n= 27)**	**P (between groups)**	**Between group Cohen’s d**
Pretreatment VAS for pain (mm) Mean±SD Median; min- max	77.3±20.0 80; 50-100	75.0±21.6 80; 40-100	75.9±20.6 80; 40-100	0.839	
Post treatment VAS for pain (mm) Mean±SD Median; min- max	40.00±27.9 40; 0-100	43.8±26.2 50; 0-90	42.2±26.5 50; 0-100	0.600	0.141
1 month post treatment VAS for pain (mm) Mean±SD Median; min- max	49.1±28.9 50; 10-100	49.1±28.9 50; 0-100	49.1±28.3 50; 0-100	0.980	0
P within groups	0.058	0.003	0.000		

*See table 1 for abbreviations*

VAS score for pain (range 0-100mm, MCII -19.9mm)

Cohen’s d 0.2= small effect size

Cohen’s d 0.5= medium effect size

Cohen’s d 0.8= large effect size

**Table 3:** WOMAC scores before and after treatment in both groups

	**Continuous** **SWD** **(n= 11)**	**Pulsed SWD** **(n= 16)**	**Total** **(n= 27)**	**P (between groups)**	**Between group** **Cohen’s d**
Pretreatment WOMAC score Mean±SD Median; min- max	52.1 ± 18.1 54; 26-78	47.9 ± 15.3 45; 16-73	49.6 ± 16.3 48; 16-78	P= 0.521	
Post treatment WOMAC score Mean±SD Median; min- max	38.4 ± 17.4 34.0; 6-65	36.9 ± 18.4 35.5; 2-71	37.5 ± 17.7 34.0; 2-71	P=0.805	0.084
1 month post treatment WOMAC score Mean±SD Median; min- max	37.4 ± 20.3 39.0; 2-62	37.4 ± 23.1 38; 0-76	37.4 ± 21.6 38; 0-76	P=0.921	0
P within groups	p=0.060	P= 0.003	P=0.000		

*See table 1 for abbreviations*

*WOMAC MCID >12% of baseline score *

Cohens d 0.2= small effect size

Cohen’s d 0.5= medium effect size

Cohen’s d 0.8= large effect size

**Table 4:** 6MWT values before and after treatment in both groups

	**Continuous** **SWD** **(n= 11)**	**Pulsed SWD** **(n=16)**	**Total** **(n=27)**	**P (between groups)**	**Between group ** **Cohen’s d**
Pretreatment 6MWT (m) Mean±SD Median; min- max	352.3±67.4 350; 250-440	323.3±88.3 327.5; 80-460	335.1 ± 80.3 340; 80-460	t=-0.920 p=0.367	
Post treatment 6MWT (m) Mean±SD Median; min- max	361.0±70.9 358; 260-500	344.4±68.8 360; 200-440	351.2±68.8 358.0; 200-500	t= -0.610 p=0.548	0.238
1 month post treatment 6MWT (m) Mean±SD Median; min- max	365.9±73.9 392; 260-483	363.6±87.8 348; 200-504	364.52±80.9 355; 200-504	t=-0.073 p=0.943	0.028
P within groups	P= 0.300	P= 0.010	P=0.041		

*See table 1 for abbreviations*

6MWT MCID 54m

Cohens d 0.2= small effect size

Cohen’s d 0.5= medium effect size

Cohen’s d 0.8= large effect size

## Discussion

The findings of this comparative effectiveness study suggest that both continuous and pulsed SWD reduce pain and improve functionality, but not walking capacity in KOA. The small effect size is in keeping with the fact that one treatment option was not found to be superior to the other. To date, published research in the literature also highlights the benefits of both CSWD and PSWD. 

The most recent systematic review and meta analysis on the treatment effects of SWD in KOA by Wang et al. has emphasized the increased reduction in pain following treatment with PSWD as opposed to CSWD (18). It is believed that the ability of PSWD to reduce inflammation and synovial thickness results in a reduction in joint stiffness and pain (28). This may explain its significant effects on pain and functionality in this study. 

In contrast, some past studies have shown that the positive effect on pain perception is achieved only when the treatment involves at least some degree of thermal sensation but that despite this, the benefits of pain reduction are lost within 9-12 weeks of follow up post therapy. Contrary to this, the study by Akyol et. al showed no extra positive effects of thermic SWD plus isokinetic exercise on pain, disability, muscle strength, walking distance in KOA when compared to exercise alone (29).

There was no clinically important improvement in walking distance as measured by the 6MWT. Laufer at al. also found no significant improvement in a three-minute walking test following treatment with thermic and athermic SWD (30). A meta-analysis conducted by the same author suggested a strong possibility of an immediate improvement on pain and functional abilities, as reported by the WOMAC questionnaire, following treatment with SWD (17).

The main limitation to the study was the small sample size. This was partly due the stringent exclusion criteria; many women with gonarthrosis in the 50-65 age range also suffer from low back and hip complaints. However, it was believed that the presence of such symptoms could confound the results and so these patients were excluded from the study. Even though the beneficial effects of SWD in the treatment of KOA are well known, many patients declined treatment with SWD. Therefore, patient recruitment may become a problem when considering the feasibility of this study on a larger scale. Even though the benefits of regular long term exercise are known, monitoring exercise compliance, especially in the long term maybe difficult. In addition, a sham SWD group can be added to future studies. 

In conclusion, the preliminary results of this pilot study suggest that treatment of KOA with both CSWD and PSWD is effective at reducing pain and improving functionality. However, a further study with a larger sample size must be performed to consolidate these findings. 
